# Alternative *Saccharomyces* interspecies hybrid combinations and their potential for low‐temperature wort fermentation

**DOI:** 10.1002/yea.3246

**Published:** 2017-08-30

**Authors:** Jarkko Nikulin, Kristoffer Krogerus, Brian Gibson

**Affiliations:** ^1^ VTT Technical Research Centre of Finland Ltd Tietotie 2, PO Box 1000 FI‐02044 VTT Espoo Finland; ^2^ Chemical Process Engineering, Faculty of Technology University of Oulu PO Box 8000 FI‐90014 Oulun Yliopisto Finland; ^3^ Department of Biotechnology and Chemical Technology Aalto University, School of Chemical Technology Kemistintie 1, Aalto, PO Box 16100 FI‐00076 Espoo Finland

**Keywords:** *saccharomyces pastorianus*, lager beer, hybridization, cold tolerance, maltotriose, aroma, phenolic off flavour

## Abstract

The lager yeast hybrid (Saccharomyces cerevisiae × Saccharomyces eubayanus) possesses two key characteristics that are essential for lager brewing: efficient sugar utilization and cold tolerance. Here we explore the possibility that the lager yeast phenotype can be recreated by hybridizing S. cerevisiae ale yeast with a number of cold‐tolerant Saccharomyces species including Saccharomyces arboricola, Saccharomyces eubayanus, Saccharomyces mikatae and Saccharomyces uvarum. Interspecies hybrids performed better than parental strains in lager brewing conditions (12°C and 12°P wort), with the S. mikatae hybrid performing as well as the S. eubayanus hybrid. Where the S. cerevisiae parent was capable of utilizing maltotriose, this trait was inherited by the hybrids. A greater production of higher alcohols and esters by the hybrids resulted in the production of more aromatic beers relative to the parents. Strong fermentation performance relative to the parents was dependent on ploidy, with polyploid hybrids (3n, 4n) performing better than diploid hybrids. All hybrids produced 4‐vinyl guaiacol, a smoke/clove aroma generally considered an off flavour in lager beer. This characteristic could however be eliminated by isolating spore clones from a fertile hybrid of S. cerevisiae and S. mikatae. The results suggest that S. eubayanus is dispensable when constructing yeast hybrids that express the typical lager yeast phenotype. © 2017 The Authors. *Yeast* published by John Wiley & Sons, Ltd.

## Introduction

Traditionally, lager beers are brewed with *Saccharomyces pastorianus* (Gibson and Liti, 2015). This bottom‐fermenting yeast is an interspecies hybrid of Saccharomyces cerevisiae and *Saccharomyces eubayanus* (Libkind *et al*., 2011). This hybridization presumably occurred in Bavaria during the fifteenth or sixteenth centuries, coincident with the rise of low‐temperature brewing of lager beer (Meussdoerffer, 2009). The rise of *S. pastorianus* to its central role in the brewing industry is presumably due to a fortuitous combination of circumstance and its superior biological properties: the ability to efficiently ferment wort sugars, which has been obtained from a S. cerevisiae parent, and cold tolerance, which has been obtained from a *S. eubayanus* parent (Gibson *et al*., 2013). The recent creation of *de novo* lager yeast hybrids has revealed that hybrid strains typically outperform parent strains during wort fermentation and that these hybrids grow well at the lower temperatures associated with lager brewing (Krogerus *et al*., 2015; Mertens *et al*., 2015). The lager yeasts that are used today in the brewing industry are closely related and exhibit little genetic or phenotypic diversity (Dunn and Sherlock, 2008; Okuno *et al*., 2016). Genetic and functional diversity could potentially be increased through the creation of *de novo* lager yeast hybrids from diverse lineages in the *Saccharomyces* genus, as long as the requisite characteristics are present.

*S. eubayanus* is not the only cold tolerant species in the *Saccharomyces* genus. Strains of *Saccharomyces kudriavzevii* and *Saccharomyces uvarum* are also adept at growing and fermenting at low temperatures (Gonçalves *et al*., 2011; González *et al*., 2006; López‐Malo *et al*., 2013; Masneuf‐Pomarède *et al*., 2010; Paget *et al*., 2014). These species are usually associated with wine and cider fermentation (Naumov *et al*., 2001; González *et al*., 2006). Hybrids of these, i.e. S. cerevisiae *× S. kudriavzevii* and S. cerevisiae *× S. uvarum*, have also been isolated from both brewing and wine‐making environments (Pérez‐Torrado *et al*., 2017; Sampaio and Gonçalves, 2008). As these ‘alternative’ *Saccharomyces* species are also cold tolerant, they may be feasible alternatives to *S. eubayanus* in the creation of interspecific hybrids for the purpose of lager brewing. The potential of *de novo* interspecific hybrids created from other species in the *Saccharomyces* genus (i.e. *Saccharomyces arboricola*, *S. kudriavzevii*, *Saccharomyces mikatae*, *Saccharomyces paradoxus* or *S. uvarum*) for brewing purposes has, to our knowledge, not been explored. However, their potential in winemaking conditions has been demonstrated in recent studies. The use of *de novo* S. cerevisiae interspecific hybrids with *S. kudriavzevii* (Bellon *et al*., 2011; Lopandic *et al*., 2016), *S. mikatae* (Bellon *et al*., 2013), S. paradoxus (Bellon *et al*., 2011) and *S. uvarum* (Bellon *et al*., 2011; Lopandic *et al*., 2016) has revealed their potential for increasing aromatic diversity and fermentation performance.

While cold tolerance probably played an important role in the emergence of lager yeast, its success can also be attributed to its fermentation properties. These include the efficient use of wort sugars (mainly maltose and maltotriose) and the production of desirable aroma compounds (Gibson and Liti, 2015). To what extent these features are attributable to the two parent species is not completely understood. Studies on the maltose and maltotriose uptake capabilities of the non‐S. cerevisiae species in the *Saccharomyces* complex are limited, but in general maltose and particularly maltotriose use tends to be poor in non‐domesticated strains (Bell *et al*., 2001; Gallone *et al*., 2016). The distribution of sugar transporters in natural lager yeasts suggests that the majority of them have been obtained from the S. cerevisiae parent. While it has been hypothesized that the maltotriose‐transporting Mtt1 transporter in lager yeast is derived from the *S. eubayanus* parent (Dietvorst *et al*., 2005; Nakao *et al*., 2009), recent studies have found this transporter in S. cerevisiae strains (Magalhães *et al*., 2016; Vidgren, 2010). Hence, it is possible that the sugar‐utilizing ability of the non‐S. cerevisiae parent of *de novo* lager yeast hybrids has little effect on the hybrid's sugar utilization abilities, as long as the S. cerevisiae parent is capable of efficient maltose and maltotriose uptake.

One important feature of lager yeast is their inability to produce 4‐vinyl guaiacol (Mukai *et al*., 2014; Richard *et al*., 2015). This compound contributes to so‐called phenolic off‐flavour (POF) and is considered undesirable in most beer styles (Gallone *et al*., 2016; Gonçalves *et al*., 2016). 4‐Vinyl guaiacol is derived from ferulic acid and is usually associated with non‐domesticated yeasts. In nature such hydroxycinnamic acid transformations serve a protective function (Stratford *et al*., 2007), but are unlikely to be important for survival in the brewing environment. The phenotype is attributed to the action of the adjacent *PAD1* and *FDC1* genes, and we have found it to be a feature of the type‐strains of all non‐S. cerevisiae *Saccharomyces* species, including *S. eubayanus*. *De novo* lager yeast hybrids created in recent studies have all produced 4‐vinyl guaiacol owing to inheritance of functional *PAD1* and *FDC1* genes from *S. eubayanus* (Krogerus *et al*., 2015; Mertens *et al*., 2015). However, if one of the parent strains contains loss‐of‐function mutations in either *PAD1* or *FDC1*, and the hybrid is also fertile*,* the phenotype can be removed through meiotic recombination and sporulation, as demonstrated by Krogerus *et al*. (2017). This suggests that flavour profile can be manipulated by accentuating positive flavours through strain selection and ploidy control and eliminating negative flavours through sporulation and selection of spore clones expressing the desired phenotype.

In this study, we explored the possibility of mating alternative *Saccharomyces* species with S. cerevisiae ale strains for the creation of new interspecies yeast hybrids suitable for lager brewing. The parents selected included two that have been associated with fermentation, in either pure (*S. uvarum*) or hybrid form (*S. eubayanus* and *S. uvarum*) and two which have never been found in a fermentation environment or exploited for any biotechnological application (S. arboricola and *S. mikatae*). Fermentation performance in lager brewing conditions, beer aroma profile and POF phenotype were assessed for all parents and hybrids.

## Materials and methods

### Yeast strains

The purpose of the work was to study the application of various interspecific *Saccharomyces* hybrids for lager beer brewing. Two ale strains from the VTT culture collection were included as parent strains: VTT‐A81062 and VTT‐A94132. The ‘alternative’ *Saccharomyces* species were represented by their type strains as follows: S. arboricola VTT‐C15952 (CBS 10644), *S. eubayanus* VTT‐C12902 (CBS 12357), *S. mikatae* VTT‐C15949 (CBS 8839) and *S. uvarum* VTT‐C05774 (CBS 395). The yeast strains used in the study are listed in Table 1. Additional information can be obtained from the VTT Culture Collection (culturecollection.vtt.fi)

**Table 1:** Yeast strains used in the study

Code	Species	Information	Ploidy	POF	Maltotriose fermentation	Source
A81062	Saccharomyces cerevisiae	Ale strain	1.99 (±0.20)	+	+	VTT Culture Collection
A94132	S. cerevisiae	Ale strain	3.93 (±0.21)	−	−	VTT Culture Collection
C15952	*Saccharomyces arboricola*	Type strain (CBS 10644)	1.99 (±0.16)	+	−	VTT Culture Collection
C12902	*Saccharomyces eubayanus*	Type strain (CBS 12357)	1.86 (±0.20)	+	−	VTT Culture Collection
C15949	*Saccharomyces mikatae*	Type strain (CBS 8839)	1.95 (±0.07)	+	−	VTT Culture Collection
C05774	*Saccharomyces uvarum*	Type strain (CBS 395)	2.03 (±0.08)	+	−	VTT Culture Collection
H1	*S. cerevisiae* × *S. eubayanus*	A81062 × C12902	2.79 (±0.25)	+	+	Krogerus *et al*. (2015)
JN2	*S. cerevisiae* × S. arboricola	A81062 × C15952	2.88 (±0.12)	+	+	This study
JN4	*S. cerevisiae* × *S. uvarum*	A81062 × C05774	1.95 (±0.12)	+	+	This study
JN5	*S. cerevisiae* × *S. uvarum*	A81062 × C05774	3.85 (±0.21)	+	+	This study
JN6	*S. cerevisiae* × *S. mikatae*	A81062 × C15949	1.93 (±0.09)	+	+	This study
JN7	*S. cerevisiae* × *S. mikatae*	A81062 × C15949	2.91 (±0.15)	+	+	This study
JN10	*S. cerevisiae* × *S. mikatae*	A94132 × C15949	4.36 (±0.19)	+	−	This study
JN11	*S. cerevisiae* × *S. mikatae*	A94132 × C15949	5.36 (±0.35)	+	−	This study
JN10/D1	*S. cerevisiae* × *S. mikatae*	Spore clone of JN10	2.44 (±0.17)	−	−	This study

### Temperature tolerance

A spot plate test was carried out to test the ability of the parent strains to grow at low and high temperatures. Strains were initially cultivated as shake flask cultures for 24 h on liquid YPD medium (24°C, 120 rpm). The viable cell densities were determined by propidium iodine staining with a ChemoMetec NucleoCounter and cells were spotted on YPD agar plates at concentrations of 1 × 10^6^, 1 × 10^5^, 1 × 10^4^, 1 × 10^3^ and 1 × 10^2^ cells per 5 μL spot. Plates were incubated at 4°C for 25 days and 20 or 37°C for 3 days.

### Hybridizations and hybrid confirmation

Prior to hybridization, attempts were made to isolate *ura‐* and *lys‐*auxotrophs of all the parent strains as described previously (Krogerus *et al*., 2015) using 5‐fluoroorotic acid (Boeke *et al*., 1987) and *α*‐aminoadipic (Zaret and Sherman, 1985) media, respectively. For hybridization, a rare‐mating technique was used. The parent strains carrying complementary auxotrophic markers were grown separately overnight in 25 mL of YPD (1% yeast extract, 2% peptone, 4% glucose). Aliquots of 1 mL of cell cultures were pelleted and washed with sterile deionized H_2_O. The pellets were resuspended in sterile, deionized water to a concentration of 10 mg of centrifuged yeast mass per millilitre of H_2_O, after which 80 μL aliquots of suspension from both parent strains were spread directly on an SD agar (0.67% Yeast Nitrogen Base without amino acids, 2% glucose, 2% agar). Colonies that emerged were considered potential hybrids, and these were first transferred to fresh SD agar, and then to YPD agar. We were unable to obtain stable auxotrophs of S. arboricola C12952, and instead utilized its inability to grow at 37°C for selection. Hybrid JN2 was obtained essentially as described above, but the wild‐type S. arboricola C12952 and a *ura‐*isolate of S. cerevisiae A81062 were used as parent strains. Hybrid selection was performed on SD agar, which was incubated at 37°C. The hybrid status of the potential hybrids was confirmed by PCR amplification using the species‐specific primers described by Muir *et al*. (2011) and Pengelly and Wheals (2013).

Meiotic segregants of the interspecific hybrid JN10 (Sc‐A94132 × Sm‐C15949) were obtained by first culturing in YPM medium (1% yeast extract, 2% peptone, 4% maltose) at 20°C overnight. The strain was then transferred to pre‐sporulation medium (0.8% yeast extract, 0.3% peptone, 10% glucose) at a starting OD600 of 0.3 and allowed to grow for 20 h at 20°C. The yeast was then washed with 1% potassium acetate and a thick suspension was plated onto sporulation agar (1% potassium acetate, 10 mg L^−1^ lysine and uracil, 2% agar). The yeast was allowed to sporulate for 7 days at 25°C. Meiotic segregants were obtained by dissecting tetrad ascospores after treatment with Zymolyase 100 T (US Biological, USA). Dissection was carried out on YPD agar with an MSM 400 micromanipulator (Springer, UK). From 64 spores, eight (13%) were found to be viable. Four of these spores were tested for POF status and one (JN10/D1) was found to be POF negative (Table 1).

The ploidy of all the strains used in the study was estimated by flow cytometry, essentially as described by Haase and Reed (2002). Cells were grown overnight in YPD medium (1% yeast extract, 2% peptone, 2% glucose), and ~1 × 10^7^ cells were washed with 1 mL of 50 mm citrate buffer. Cells were then fixed with cold 70% ethanol, and incubated at room temperature for 1 h. Cells were then washed with 50 mm citrate buffer (pH 7.2), resuspended in 50 mm citrate buffer containing 0.25 mg mL^−1^ RNAse A and incubated overnight at 37°C. A 1 mg mL^−1^ solution of Proteinase K was then added, and cells were incubated for 1 h at 50°C. Cells were then stained with SYTOX Green (2 μm; Life Technologies, USA), and their DNA content was determined using a FACSAria IIu cytometer (Becton Dickinson, USA). DNA contents were estimated by comparing the mean peak fluorescence intensities with those of S. cerevisiae haploid (CEN.PK113‐1A) and diploid (CEN.PK) reference strains (set to a ploidy of 1.0 and 2.0, respectively). Measurements were performed on duplicate independent yeast cultures, and 100 000 events were collected per sample during flow cytometry. Data was processed with the ‘flowCore’ package (Hahne *et al*., 2009) in R, while mean peak fluorescence intensities were estimated with the ‘normalmixEM’ function of the ‘mixtools’ package (Benaglia *et al*., 2009) in R.

### Wort fermentation and sampling

Brewer's all‐malt wort was made at VTT Technical Research Centre of Finland. Worts (15°P) were prepared with Espoo city water, collected while hot (>90°C) and stored at 0°C until use. Before use, worts were diluted to 12°P by adding autoclaved city water and aerated to 10 ppm. Wort contained maltotriose at 13.9 g L^−1^, maltose at 55.2 g L^−1^, glucose at 12.1 g L^−1^ and fructose at 4 g L^−1^.

For each fermentation, loopfuls of the yeasts were grown first in 25 mL of YPD at room temperature on a shaker (120 rpm) for 2 days before the suspensions as a whole were transferred to 500 mL of 12°P wort and incubated at 12°C on a shaker (120 rpm) for the next 2 days. The yeasts were collected and 20% slurries (*w*/w) were prepared with the spent wort. Generation 0 (‘G0’) fermentations were conducted with 2 L cylindroconical stainless steel fermenting vessels imitating industrial brewing conditions. A pitching rate of 4 g of yeast L^−1^ of wort and 1.5 L of 12°P wort were used for fermentations for 5–7 days.

For the main fermentation (‘G1’), the yeasts were once more collected and 20% slurries (*w*/w) were prepared. The viable cell densities were determined as above. Yeast was pitched at a rate of 1 × 10^7^ cells mL^−1^ of wort in 1.5 L of 12°P wort. First samples were taken 1 h after pitching and followed by regular sampling at 1–2 day intervals. Results are means of two independent experimental replicates and only values from G1 fermentations are reported.

The density, ethanol concentration and pH of the samples were determined from the centrifuged and degassed fermentation samples using an Anton Paar Density Meter DMA 5000 M with Alcolyzer Beer ME and pH ME modules (Anton Paar GmbH, Austria). The yeast pellet of the samples was washed with deionized H_2_O, transferred to pre‐weighed porcelain crucibles, dried overnight at 105°C and weighed once more to determine the dry mass content. Residual sugars and yeast‐derived aroma compounds were measured exactly as described in Krogerus *et al*. (2017). The POF phenotype of the yeast strains was assessed in a small‐scale assay as described in Krogerus *et al*. (2017). A 1 mL aliquot of YPM supplemented with 100 mg L^−1^ of trans‐ferulic acid was inoculated with a colony of each yeast strain. The cultures were incubated for 48 h at 25°C. The POF phenotype was determined sensorially by examining for the presence (POF+) or absence (POF−) of the distinct clove‐like aroma of 4‐vinyl guaiacol.

Statistical analysis was performed with R (http://www.r‐project.org/) using one‐way ANOVA and Tukey's test. Heat maps of the concentrations of yeast‐derived flavour compounds in the beers were generated in R based on *z‐*scores. The *z‐*scores (*z*) were calculated as *z* = (*x* − *μ*)/*σ*, where *x* is the concentration of an aroma compound in a particular beer, *μ* is the mean concentration of that aroma compound in all beers and *σ* is the standard deviation of concentration of that aroma compound in all beers.

## Results and discussion

In this study, we explored the potential of mating alternative *Saccharomyces* species with S. cerevisiae ale strains for the creation of interspecific yeast hybrids suitable for lager brewing. We successfully generated hybrids of S. cerevisiae and three other *Saccharomyces* species: S. arboricola, *S. mikatae* and *S. uvarum* (Figure S1 in the Supporting Information). In addition to the newly generated hybrids, we included the previously generated S. cerevisiae A81062 × *S. eubayanus* C12902 ‘Hybrid H1’ for comparison (Krogerus *et al*., 2015) during characterization.

### Parental strains differ in their tolerance to temperature extremes and ability to ferment wort at low temperature

**Figure 1:**
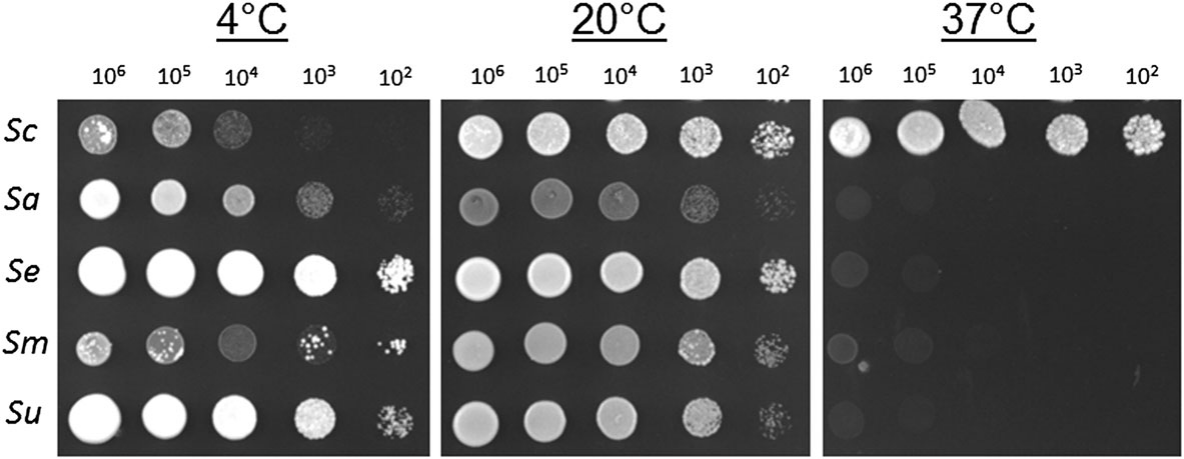
Growth of the parental strains Saccharomyces cerevisiae A81062 (*Sc*), *Saccharomyces arboricola* C15952 (*Sa*), *Saccharomyces eubayanus* C12902 (*Se*), *Saccharomyces mikatae* C15949 (*Sm*), and *Saccharomyces uvarum* C05774 (*Su*), at 4, 20 and 37°C. YPD agar cultures were incubated for 3 days at 20 and 37°C and 21 days at 4°C

A growth temperature of 37°C restricted the growth of all species except for S. cerevisiae (Figure 1). At 4°C all strains were capable of growth but this was markedly reduced in the case of S. cerevisiae. The *S. eubayanus* and *S. uvarum* strains appeared to exhibit the greatest cold tolerance, while the S. arboricola and *S. mikatae* tolerances seemed intermediate relative to the others. The results indicated that these strains (S. arboricola, *S. mikatae* and *S. uvarum*) could contribute to cold tolerance of constructed interspecies hybrids when used instead of *S. eubayanus*. This claim is supported by improved growth of interspecies hybrids at 4°C (Figure S2).

**Figure 2:**
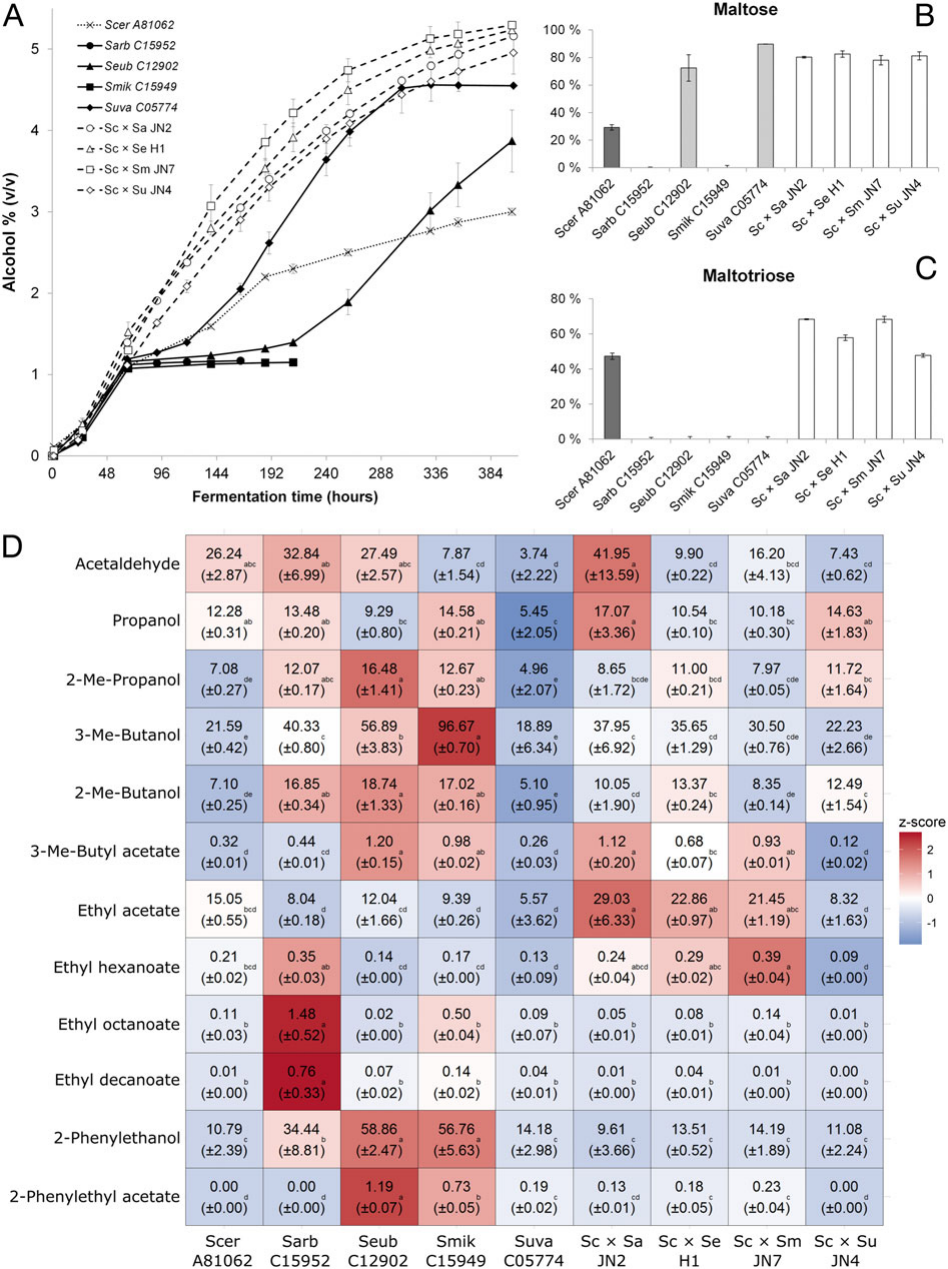
Fermentation potential of alternative hybrids and parental strains and characteristics of beers produced. (A) The alcohol content of the 12°P wort fermented at 12°C with *de novo* hybrids (open symbols) and parent strains (solid symbols). (B) The maltose and (C) maltotriose utilization (percentage of the concentration in the original wort) of the *de novo* hybrids (open bars) and their parent strains (grey bars) after fermentation. Values are means from two independent fermentations and error bars where visible represent the standard deviation. (D) Normalized concentrations (mg L^−1^) of aroma compounds (rows) in the beers fermented with *de novo* hybrids and parental strains (columns). The heat map was generated based on the *z‐*scores (blue and red indicate low and high values, respectively). Values are means from two independent fermentations (standard deviation in parentheses) and they have been normalized to an ethanol concentration of 5% (*v*/v). Different letters in rows indicate significant difference based on one‐way Anova and Tukey's test. Abbreviations: Sa and Sarb, S. arboricola; Sc and Scer, S. cerevisiae; Se and Seub, *S*. *eubayanus*; Sm and Smik, *S*. *mikatae*; Su and Suva, *S*. *uvarum*; and Me, methyl

All five parental strains performed poorly during low‐temperature wort fermentations relative to the constructed hybrids, although performance varied between strains (Figure 2A). At the beginning of fermentation up until 72 h, during which mainly glucose and fructose are consumed from the wort, all of the parental strains performed similarly. After this point, the fermentation performance of the parental strains was affected by differential utilization of wort sugars. In the cases of S. arboricola and *S. mikatae*, fermentation was limited by an apparent inability to utilize either of the major *α*‐glucosides present (Figure 2B and C). *Saccharomyces eubayanus* performance was sluggish until 8 days after pitching at which point the strain appeared to adapt to maltose utilization, with a final utilization of 75%. *Saccharomyces uvarum* fermentation was relatively rapid compared with the other parents but was limited by an inability to utilize maltotriose (Figure 2C). As in previous studies (Krogerus *et al*., 2015, 2016), performance of the S. cerevisiae parent was poor despite its ability to utilize the major wort sugars; this was presumably due to its sensitivity to low‐temperature, lager‐brewing conditions, a supposition supported by the restricted growth seen at low temperature in this study (Figure 1). Results highlight the respective deficiencies of the parental strains when assessed under these conditions, i.e. S. cerevisiae's reduced fermentative ability at low temperature and the incomplete sugar utilization of the non‐S. cerevisiae strains. Despite their inability to utilize wort maltotriose, *S. eubayanus* and *S. uvarum* demonstrated some potential for brewing. In the case of the former this potential has already been exploited for commercial beer production. The brewing potential of *S. uvarum* has, however, not been demonstrated previously. This species, although exhibiting good cold tolerance, would suffer from the same phenotypic deficiencies as *S. eubayanus*, namely inability to utilize maltotriose and production of the smoke/clove aroma 4‐vinylguaiacol (a trait observed for all the non‐S. cerevisiae strains in this study). Both features compromise the strains' use for mainstream lager brewing.

### Constructed hybrids display parental transgression in low‐temperature wort fermentation conditions

In an attempt to overcome the aforementioned deficiencies in the parent strains, interspecific hybrids were generated between the S. cerevisiae and the non‐S. cerevisiae strains through rare mating. Hybridization was performed directly on the selection agar plates, i.e. combining mating and selection (as was done for all the hybrids here except for H1), which proved to be effective. Furthermore, all of the emerging colonies isolated could be considered distinct hybrids. The hybridization frequency (~10^−7^) was still considerably lower than that which can be achieved through spore‐to‐spore mating (Mertens *et al*., 2015), but the generated hybrids varied considerably in DNA content, ranging from ~2*n* to >5*n* (Table 1). Higher ploidy can have practical advantages, e.g. improved wort fermentation (Krogerus *et al*., 2016) and allotetraploidy, which may allow for hybrid fertility (Greig *et al*., 2002; Krogerus *et al*., 2017; Sebastiani *et al*., 2002).

Fermentation performance was characterized in fermentations of 12°P wort at 12°C. Initially, hybrids (H1, JN2, JN4 and JN7) created with S. cerevisiae A81062 and each of the four non‐S. cerevisiae parents were compared with the parent strains. All four hybrids performed well throughout the fermentation, and clearly outperformed the parental strains in regards to the alcohol yield (Figure 2A). It was especially interesting to note that the S. arboricola‐ and *S. mikatae*‐derived hybrids performed well in wort, despite the parent strains not demonstrating any clear capabilities of utilizing maltose or maltotriose (Figure 2B and C). This suggests that the *α*‐glucoside uptake ability of these hybrids was inherited mainly, or exclusively, from the S. cerevisiae parent. Surprisingly, the S. cerevisiae × *S. mikatae* hybrid JN7 performed as well as the S. cerevisiae × *S. eubayanus* hybrid H1 throughout fermentation (no significant difference detected by Student's *t*‐test), despite the poor fermentation of *S. mikatae* alone. With *S*. *eubayanus*‐ and *S. uvarum*‐derived hybrids, enhanced sugar consumption could be related to either or both parental strains. There was, however, no obvious competitive advantage when compared with the other hybrids, suggesting that sugar‐utilization capabilities inherited from the non‐S. cerevisiae parents may be of secondary importance.

### Hybridization impacts beer aroma profile

Apart from the increase in fermentation performance and the more complete sugar use by the hybrids compared with the parent strains, an increased formation of desirable aroma‐active esters was also observed in the hybrid strains (Figure 2D). These were 3‐methylbutyl acetate (banana aroma), ethyl hexanoate (apple aroma) and ethyl octanoate (fruity aroma) in particular, where overall higher levels were observed in the hybrids compared with the parent strains. Here, all values were normalized to 5% alcohol (*v*/v) in order to aid comparison between the hybrid strains. The values for the low‐ethanol parent strains S. arboricola and *S. mikatae* are artificially high owing to this normalization (the original pre‐normalized concentrations are presented in Table S1 in the Supporting Information). Interestingly, the S. arboricola‐derived hybrid JN2 (Sc‐A81062 × Sa‐C15952) produced the highest concentrations of 3‐methylbutyl acetate among the hybrids, despite the S. arboricola and S. cerevisiae parent strains producing low concentrations of it when compared with the other parent strains. Similarly, the *S. mikatae*‐derived hybrid JN7 produced the highest concentrations of ethyl hexanoate, despite the *S. mikatae* parent producing low concentrations compared with the other parent strains. The production of aroma‐active esters and higher alcohols in *Saccharomyces* yeasts is affected by both environmental and genetic factors (Pires *et al*., 2014; Steensels *et al*., 2014). Studies have shown that aroma formation can vary significantly within and among different yeast species (Steensels *et al*., 2014; Gallone *et al*., 2016; Gamero *et al*., 2016), and this diversity could be exploited for developing strains suitable for the lager brewing industry. By expanding the range of parent strains to species other than S. cerevisiae and *S. eubayanus*, one could further diversify the limited aroma spectrum that exists in natural lager yeast (Mertens *et al*., 2015). Furthermore, as in many previous studies (Bellon *et al*., 2011; Steensels *et al*., 2014; Mertens *et al*., 2015; Krogerus *et al*., 2016), we observed heterosis for several aroma compounds, which allows for generation of strains producing unique and strong aroma profiles. Here, we studied only a limited set of aroma compounds, and it is likely that more variation between the parent strains and the hybrids could be observed in other undetected aroma compounds.

### Ploidy of interspecies hybrids influences functional traits

**Figure 3:**
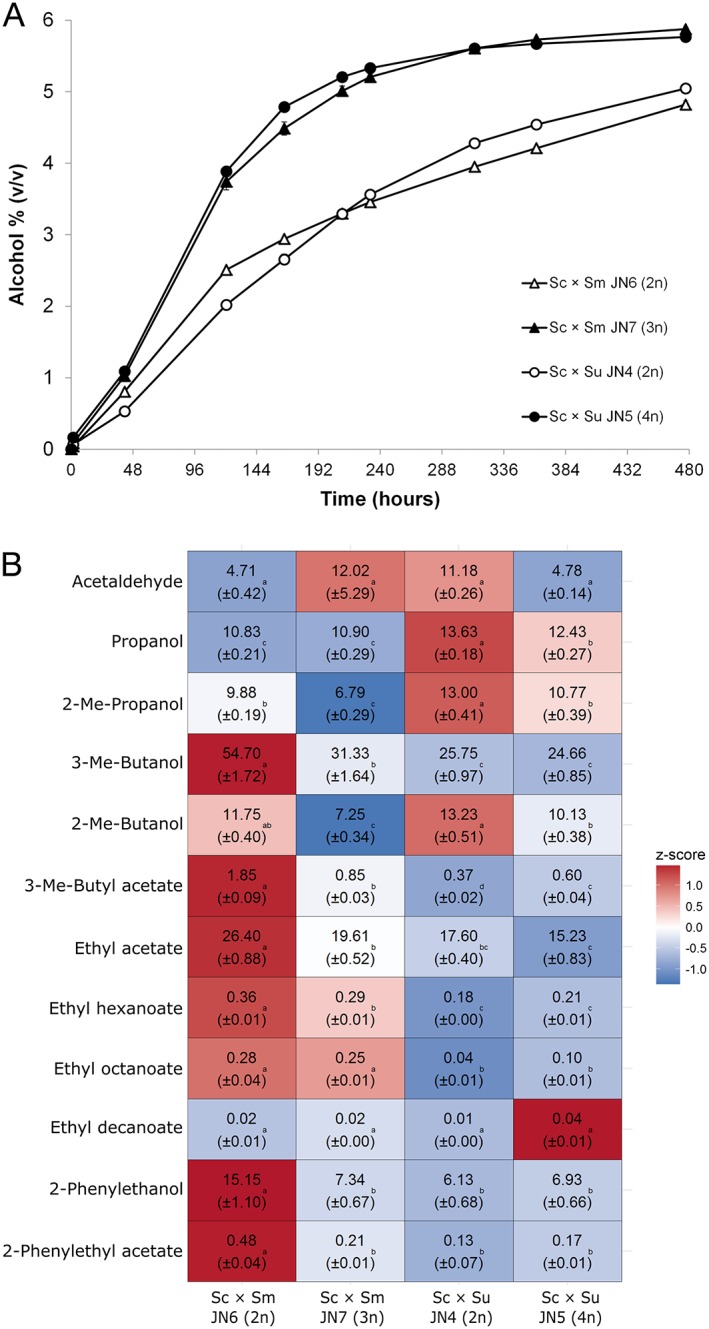
Fermentation potential of *de novo* hybrids of different ploidy and aroma content of resultant beers. (A) The alcohol content of the 12°P wort fermented at 12°C. Diploid and polyploid hybrids are represented by open and solid symbols, respectively. Values are means from two independent fermentations and error bars where visible represent the standard deviation. (B) Normalized concentrations (mg L^−1^) of aroma compounds (rows) in the beers. The heat map was generated based on the *z‐*scores (blue and red indicate low and high values, respectively). Values are means from two independent fermentations (standard deviation in parentheses) and they have been normalized to an ethanol concentration of 5% (*v*/v). Different letters in rows indicate significant difference based on one‐way Anova and Tukey's test. Abbreviations: Sc, S. cerevisiae; Sm, *S. mikatae*; Su, *S. uvarum*; and Me, methyl

As seen in other studies (Krogerus *et al*., 2016; Mertens *et al*., 2015), the ploidy of lager hybrids may play a role in fermentation performance. These studies revealed that higher DNA content may contribute to improved fermentation performance and greater aroma production. Here, the effects of ploidy of alternative *de novo* hybrids were investigated. The hybrids included here for comparison were the S. cerevisiae × *S. mikatae* hybrids JN6 and JN7, a diploid and a triploid, respectively, and S. cerevisiae × *S. uvarum* hybrids JN4 and JN5, a diploid and a tetraploid, respectively. All crosses were made using a rare mating technique in which the expected outcome is a tetraploid hybrid generated by the rate mating of two diploid cells. The different ploidies seen in strains here is probably due to the presence of spontaneously formed spores during hybridization, leading to spore to spore mating (diploid hybrids) and spore to cell mating (triploid hybrids). Results demonstrate a clear positive effect of higher DNA content on fermentation performance. Both JN5 and JN7 outcompeted the corresponding diploid strains with regards to alcohol production during the fermentation (Figure 3A). The chromosome distribution and subgenome ratios of these hybrids were not investigated here, but they are expected to affect how the hybrids perform. The triploid JN7, for example, presumably contains two copies of S. cerevisiae chromosomes and one copy of *S. mikatae* chromosomes, as a result of the poor sporulation efficiency of the A81062 strain. An additional copy of the *cerevisiae*‐derived chromosomes could also explain the better performance of JN7 compared with JN6, as *S. mikatae* performed poorly in wort. Despite the relatively poor performance of the diploid hybrids, they have the ability to utilize sugars that are not accessible to the parent species *S. mikatae* (both maltose and maltotriose) and *S. uvarum* (maltotriose). Attenuation levels of the JN6 and JN4 hybrids were 72 and 77%, respectively at the time the fermentation was stopped. In previous fermentations the parental strains *S. mikatae* and *S. uvarum* were unable to achieve attenuation levels >10 and 68%, respectively.

Ploidy and subgenome inheritance also appeared to affect the aroma compounds produced by these hybrids (Figure 3B). When comparing the aroma profiles of the S. cerevisiae × *S. mikatae* hybrids JN6 and JN7, it was seen that the concentrations of many higher alcohols, 3‐methylbutyl acetate, 2‐phenylethanol and 2‐phenylethyl acetate were higher in the diploid JN6 compared with the triploid JN7 (Figure 3B and Table S2 in Supporting Information). These compounds are also produced in higher concentrations by the *S. mikatae* parent compared with the S. cerevisiae parent (Figure 2D). Our previous work with S. cerevisiae × *S. eubayanus* hybrids (Krogerus *et al*., 2016) revealed that aroma formation in hybrids is affected by subgenome inheritance and ratios. As the ratio between subgenomes in JN6 is 1:1, the aroma results would also support our assumption that JN7 contains a diploid S. cerevisiae genome. There was less difference between the S. cerevisiae × *S. uvarum* hybrids JN4 and JN5, but there appeared to be a slight increase in ester concentrations in the beer made with the tetraploid JN5. The results highlight the possibility of introducing variation not only by expanding the range of parent strains to species other than S. cerevisiae and *S. eubayanus*, but also by affecting the ploidy and how much of the parental genomes are transferred to the hybrids.

### Parental strain choice influences fermentation properties of hybrids

**Figure 4:**
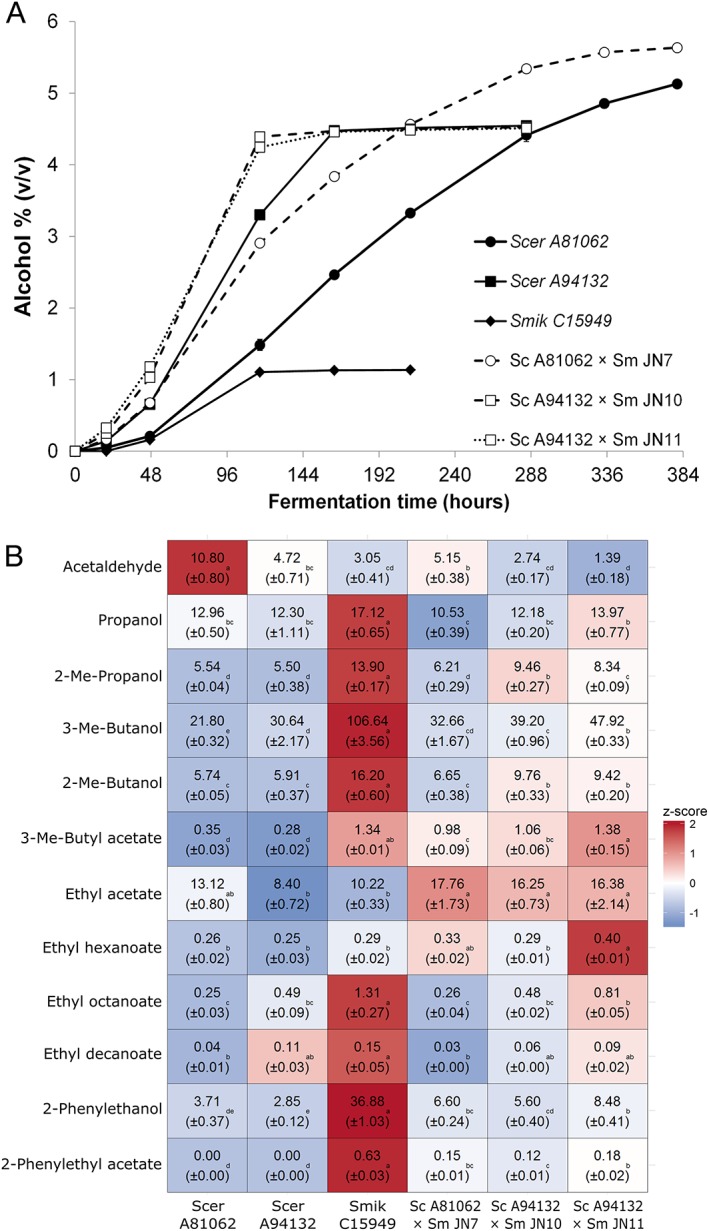
Fermentation potential of S. cerevisiae × *S. mikatae hybrids*. (A) The alcohol content of the 12°P wort fermented at 12°C with *de novo* hybrids (open symbols) and parent strains (solid symbols). Values are means from two independent fermentations and error bars where visible represent the standard deviation. (B) Normalized concentrations (mg L^−1^) of aroma compounds (rows) in the beers fermented with *de novo* S. cerevisiae × *S. mikatae* hybrids and parental strains (columns). The heat map was generated based on the *z‐*scores (blue and red indicate low and high values, respectively). Values are means from two independent fermentations (standard deviation in parentheses) and they have been normalized to an ethanol concentration of 5% (*v*/v). Different letters in rows indicate significant difference based on one‐way Anova and Tukey's test. Abbreviations: Scer, S. cerevisiae; Sm and Smik, *S*. *mikatae*; and Me, methyl

Because the *S. mikatae*‐derived hybrid JN7 and the *S. eubayanus*‐derived hybrid H1 performed similarly during fermentation and produced beers with similar aroma profiles, despite the parent strains showing clear differences, we decided to further investigate *S. mikatae* as a surrogate for *S. eubayanus* in lager hybrids. As the phenotypes between strains within a species may vary greatly (Gallone *et al*., 2016; Gonçalves *et al*., 2016), we generated additional hybrids with another S. cerevisiae parent, A94132. This strain was chosen as it possesses a phenotype that is quite different from the A81062 S. cerevisiae strain. A94132 is POF‐negative and maltotriose‐negative, while A81062 is positive for both traits. To compare the influence of the parental strains, we carried out a set of wort fermentations with the A94132 × *S. mikatae* hybrids JN10 and JN11, the A81062 × *S. mikatae* hybrid JN7 and the parental strains (Figure 4A). Fastest fermentation rates were seen with the A94132‐derived hybrids. However, alcohol production was limited by their inability to utilize maltotriose and apparent attenuation did not exceed 68%. Hybrid JN7, derived from S. cerevisiae A81062, reached the attenuation level of 68% in ~9 days and a final attenuation level of 86% in 16 days. Parental strain performance was characterized by limited sugar utilization ability relative to the constructed hybrids.

Aroma profiles of the hybrids (Figure 4B and Table S3 in Supplementary material) again revealed that mid‐parent or best‐parent values of many desirable aroma‐active esters could be achieved using the hybrid strains compared with the parent strains. The aroma profiles of the hybrids also seemed to be dependent, to some extent, on the S. cerevisiae parent strain. Hybrid JN7 (Sc‐A81062 × Sm‐C15949) and its S. cerevisiae parent tended to produce high ethyl acetate concentrations, while Hybrids JN10 and JN11, and their parent strain A94132, were high producers of ethyl octanoate and ethyl decanoate. Genome compositions of these hybrids were not studied here, but it is likely that JN11, based on ploidy, contains an additional copy of the *S. mikatae*‐derived chromosomes compared with JN7 and JN10. This could explain the higher concentrations of the *S. mikatae*‐related aroma compounds 3‐methylbutyl acetate, 2‐phenylethanol and 2‐phenylethyl acetate in the beer fermented with hybrid JN11.

### The POF phenotype may be eliminated through spore clone selection

All hybrids considered thus far were found to be POF+ (Table 1). The phenolic taste and aroma imparted by 4‐vinyl guaiacol is typically deemed unpleasant in beers, particularly lager beers, which are associated with clean flavour profiles. Loss of 4‐vinyl guaiacol production is a signature of domestication in brewing yeast (Gonçalves *et al*., 2016; Gallone *et al*., 2016) and most ale strains and all lager yeast strains are POF−. The POF phenotype was apparently removed from brewing strains through artificial selection and may have occurred simply through brewers discarding yeast batches that produced excessive 4‐vinyl guaiacol and retaining those that produced a cleaner tasting beer. Recently constructed S. cerevisiae × *S. eubayanus* hybrids have all produced 4‐vinyl guaiacol (Krogerus *et al*., 2015; Mertens *et al*., 2015) through inheritance of functional *PAD1* and *FDC1* genes from *S. eubayanus*. This characteristic restricts the hybrids' potential for mainstream lager brewing and elimination of the trait would therefore be beneficial.

**Figure 5:**
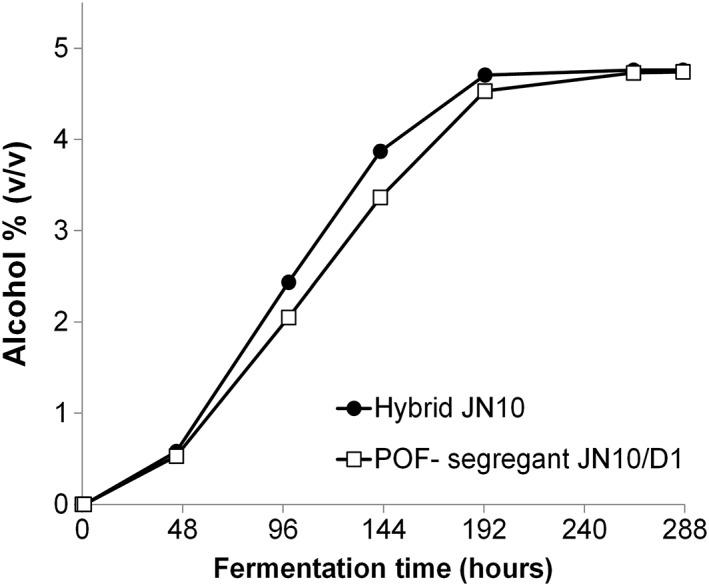
The alcohol content of the 12°P wort fermented at 12°C with Hybrid JN10 (solid circles) and its POF‐segregant JN10/D1 (open circles). Values are means from two independent fermentations and error bars where visible represent the standard deviation

Previous studies have shown that the POF+ phenotype can be removed through sporulation of intraspecific hybrids, where one of the original parents was positive for the trait and one negative (Tubb *et al*., 1981; Gallone *et al*., 2016). The segregation of chromosomes that occurs during meiosis results in some spores that inherit only the non‐functional forms of *PAD1* or *FDC1*. Elimination of the trait in the same way from interspecies hybrids is complicated by their sterility. However, as fertility may be recovered in tetraploid strains, rare mating can facilitate this approach. Krogerus *et al*. (2017) demonstrated the potential for phenotype selection through the use of fertile tetraploid intermediates. This approach was successfully used to generate S. cerevisiae × *S. eubayanus* strains that possessed multiple beneficial traits of the parents (psychrotolerance, maltotriose use, clean flavour profile; Krogerus *et al*., 2017). The A81062 ale strain used in the current study has retained the POF+ phenotype, and hybrids created with this strain are therefore not suitable for generation of POF− spore clones. However, such spore clones could be generated through sporulation of the JN10 hybrid (Sc‐A94132 × Sm‐C15949), where the ale strain involved was POF−. This polyploid strain was found to be semi‐fertile (13% spore viability) and of four spore clones derived from this strain, one was found to not produce 4‐vinyl guaiacol. A direct comparison of the brewing performance of the original hybrid and the spore clone revealed only a slight reduction in fermentation rate in mid fermentation (Figure 5), possibly as a consequence of the lower ploidy of the spore clone. Alcohol yield was unaffected and only minor differences in aroma profile (acetaldehyde, higher alcohols and esters) were observed (Table 2). The results suggest that the POF+ character of non‐domesticated yeasts is not an impediment to their industrial exploitation for lager brewing as the trait can be removed via hybridization and sporulation.

**Table 2:** Concentrations (mg L^−1^) of aroma compounds in the beers produced with Hybrid JN10 and its POF‐segregant JN10/D1

Compound	Hybrid JN10	POF‐ segregant JN10/D1
Acetaldehyde	2.2 (± 0.14)	4.0 (± 0.21)*
Propanol	10.1 (± 0.49)	8.0 (± 0.11)*
2‐Methylpropanol	7.5 (± 0.48)	4.2 (± 0.08)*
3‐Methylbutanol	38.6 (± 2.08)	38.1 (± 0.82)
2‐Methylbutanol	8.3 (± 0.47)	7.6 (± 0.17)
3‐Methybutyl acetate	1.06 (± 0.06)	1.13 (± 0.04)
Ethyl acetate	14.4 (± 0.86)	15.8 (± 0.36)*
Ethyl hexanoate	0.35 (± 0.02)	0.37 (± 0.01)
Ethyl octanoate	0.19 (± 0.02)	0.07 (± 0.003)*
Ethyl decanoate	0.024 (± 0.003)	0.004 (± 0.001)*
2‐Phenylethanol	4.6 (± 0.54)	4.2 (± 0.06)
2‐Phenylethyl acetate	0.18 (± 0.02)	0.13 (± 0.02)*

* Significant change (*p* < 0.05) in JN10/D1 compared with JN10.

### Alternative saccharomyces interspecies hybrids: Potential and perspective

Our hypothesis that cold‐tolerant *Saccharomyces* yeast, including S. arboricola, *S*. *mikatae* and *S. uvarum*, can potentially be used instead of *S. eubayanus* in the creation of *de novo* lager yeast strains is supported by the results obtained. The superior psychrotolerance of *S. eubayanus* relative to the other non‐S. cerevisiae yeast did not translate to superior fermentation performance of the *S. eubayanus* hybrids relative to the alternative hybrid strains. Cold tolerance of S. arboricola, *S. mikatae* and *S. uvarum* appears to be sufficient for the production of hybrid strains that perform well under standard lager brewing conditions. Strong fermentation performance was seen in hybrids created with the parental species S. arboricola and *S. mikatae*, despite the parents' inability to utilize the main wort sugars. This suggests that efficient maltose utilization of *S. eubayanus* is not necessarily an important factor in the success of lager brewing yeast (either traditional *S. pastorianus* strains or more recently created S. cerevisiae × *S. eubayanus* hybrids).

It may be speculated that the success of *S. pastorianus* was due to the prohibition in Bavaria in 1533 of brewing in the warmer months of the year (an event that coincided with the advent of lager brewing; Dornbusch, 1997). Lower‐temperature brewing would be expected to favour psychrotolerant yeasts, particularly those with an ability to utilize wort sugars. The conditions were therefore favourable for hybridization of an S. cerevisiae ale strain with a more cold‐tolerant species. The apparent dispensability of *S. eubayanus* for expression of the lager brewing phenotype raises the question of why this species in particular formed the original, crucial alliance with S. cerevisiae. Was the hybridization event merely a result of historical happenstance or did *S. eubayanus* have a competitive advantage over other *Saccharomyces* species, perhaps because of superior cold tolerance and maltose utilization, allowing it to proliferate in brewers wort to such an extent that a hybridization with S. cerevisiae became more likely? It could also be the case that *S. eubayanus* was more common in Europe in the past and may have been a frequent contaminant of brewery fermentations at the advent of lager brewing. Its apparent absence or extreme scarcity in modern times could be a result of deforestation or other form of habitat loss. Alternatively, the methods used for sampling to date may have been biased towards mesophilic strains. Sample enrichment at temperatures of 10°C or lower may be necessary for isolation of psychrophilic yeasts (Sampaio and Gonçalves, 2008). It may also have been the case that the selection conditions in breweries at the time of the original hybridization were more extreme than the 12°C used for assessment of hybrids here. It is conceivable that, without temperature control, fermentation temperatures could have dropped below 4°C, possibly favouring hybrids involving the most psychrotolerant parents. Other factors such as tolerance to osmotic stress, ethanol toxicity and resistance to the antimicrobial effects of hop compounds may also have played a role in the success of *S. eubayanus*. These characteristics are all awaiting comparative characterization for members of the *Saccharomyces* genus, and this information is required before definitive conclusions can be reached.

While the use of *S. mikatae* or its hybrids in brewing environments has not been explored previously, recent studies have shown the potential of S. cerevisiae × *S. mikatae* hybrids in winemaking (Bellon *et al*., 2013). There, hybrids showed broader ranges of temperature tolerance compared with the parent strains, and wines fermented with these hybrids had more complex aroma profiles. Recently Peris *et al*. (2017) also indicated the potential of *S. mikatae*, in a hybrid partnership with S. cerevisiae, for application in second‐generation bioethanol fermentation, in that case owing to its natural ability to withstand the toxic effects of lignocellulosic hydrolysates. These investigations suggest that interspecific hybridization is a potentially valuable way to exploit yeast diversity for industrial applications, particularly where species, like *S. mikatae*, may have previously been overlooked owing to their limited fermentation performance. In addition, *S. eubayanus* is geographically restricted and recreation of the lager yeast phenotype with alternative species may be more practical in, for example, Europe where other species, such as *S. uvarum*, are more accessible. It remains to be seen if other cold‐tolerant species, such as *S. kudriavzevii* and the apparently abundant European species S. paradoxus (Brysch‐Herzberg and Seidel, 2017) could also be substituted for *S. eubayanus*, and further investigation is clearly required. Despite this, these initial results suggest that the use of alternative parents for the creation of new lager brewing strains has potential to introduce genetic, and presumably phenotypic, diversity to the lager yeast group, thereby permitting the creation of more diverse beers. Selection of specific parental strains and hybridization techniques, as well as evolutionary engineering approaches that take advantage of the instability of hybrid genomes, are all expected to have an influence on the phenotypic outcome of alternative hybrid crosses and will allow design of production strains to match specific product and process specifications.

## Conflict of interest

The authors declare that there is no conflict of interest.

## Supporting information


**Table S1.** Concentrations (mg L^−1^) of aroma compounds in the beers produced with the *de novo* hybrids and parent strains. Values are means from two independent fermentations (standard deviation in parentheses). These values were used to generate the heatmap in Figure 4 in the main text after normalization to ethanol concentration.
**Table S2.** Concentrations (mg L^−1^) of aroma compounds in the beers produced with the *de novo* hybrids of different ploidy. Values are means from two independent fermentations (standard deviation in parentheses). These values were used to generate the heatmap in Figure 6 in the main text after normalization to ethanol concentration.
**Table S3.** Concentrations (mg L^−1^) of aroma compounds in the beers produced with the *de novo* S. cerevisiae × *S. mikatae* hybrids and their parent strains. Values are means from two independent fermentations (standard deviation in parentheses). These values were used to generate the heatmap in Figure 8 in the main text after normalization to ethanol concentration.
**Figure S1.** Hybrid confirmation by species‐specific PCR. Strains included are the parental strains S. cerevisiae A81062 and A94132, S. arboricola C15952, *S. eubayanus* C12902, *S. mikatae* C15949 and *S. uvarum* C05774 along with the interspecies hybrids JN2 (A81062 x C15952), H1 (A81062 x C12902), JN10 (A94132 x C15949) and JN5 (A81062 x C05774). Genes amplified are *MEX6*7/YPL169C (S. cerevisiae), *SEC24*/YIL109C (S. arboricola), *FSY1* (*S. eubayanus*) and *DBP6*/YNR038W (*S. uvarum*). All hybrids with the same parental combinations showed identical banding patterns.
**Figure S2.** Growth of the parental strain S. cerevisiae A81062 (*Sc*), hybrid JN2 (S. cerevisiae A81062 x S. arboricola C15952), hybrid H1 (A81062 x *S. eubayanus* C12902), hybrid JN7 (A81062 x *S. mikatae* C15949), and hybrid JN4 (A81062 x *S. uvarum* C05774). YPD agar cultures were incubated 21 days at 4°C.

## References

[yea3246-bib-0001] Bell PJL , Higgins VJ , Attfield PV . 2001 Comparison of fermentative capacities of industrial baking and wild‐type yeasts of the species *Saccharomyces cerevisiae* in different sugar media. Lett Appl Microbiol 32: 224–229. 11298930 10.1046/j.1472-765x.2001.00894.x

[yea3246-bib-0002] Bellon JR , Eglinton JM , Siebert TE , *et al* 2011 Newly generated interspecific wine yeast hybrids introduce flavour and aroma diversity to wines. Appl Microbiol Biotechnol 91: 603–612. 21538112 10.1007/s00253-011-3294-3

[yea3246-bib-0003] Bellon JR , Schmid F , Capone DL , *et al* 2013 Introducing a new breed of wine yeast: interspecific hybridisation between a commercial *Saccharomyces cerevisiae* wine yeast and *Saccharomyces mikatae* fairhead C. (ed). PLoS One 8 e62053. 10.1371/journal.pone.0062053 PMC3629166 23614011

[yea3246-bib-0004] Benaglia T , Chauveau D , Hunter DR , Young D . 2009 mixtools: An *R* package for analyzing finite mixture models. J Stat Softw 32: 1–29.

[yea3246-bib-0005] Boeke JD , Trueheart J , Natsoulis G , Fink GR . 1987 5‐Fluoroorotic acid as a selective agent in yeast molecular genetics. Methods Enzymol 154: 164–175. 3323810 10.1016/0076-6879(87)54076-9

[yea3246-bib-0006] Brysch‐Herzberg M , Seidel M . 2017 Distribution patterns of *Saccharomyces* species in cultural landscapes in Germany. FEMS Yeast Res. https://doi.org/10.1093/femsyr/fox033. 10.1093/femsyr/fox033 28520895

[yea3246-bib-0007] Dietvorst J , Londesborough J , Steensma HY . 2005 Maltotriose utilization in lager yeast strains: *MTT1* encodes a maltotriose transporter. Yeast 22: 775–788. 16088872 10.1002/yea.1279

[yea3246-bib-0008] Dornbusch H . 1997 Prost! The Story of German Beer. Siris Books: Boulder, Colorado, USA.

[yea3246-bib-0009] Dunn B , Sherlock G . 2008 Reconstruction of the genome origins and evolution of the hybrid lager yeast *Saccharomyces pastorianus* . Genome Res 18: 1610–1623. 18787083 10.1101/gr.076075.108 PMC2556262

[yea3246-bib-0010] Gallone B , Steensels J , Prahl T , *et al* 2016 Domestication and divergence of *Saccharomyces cerevisiae* beer yeasts. Cell 166: 1397–1410. e16. 27610566 10.1016/j.cell.2016.08.020 PMC5018251

[yea3246-bib-0011] Gamero A , Quintilla R , Groenewald M , Alkema W , Boekhout T , Hazelwood L . 2016 High‐throughput screening of a large collection of non‐conventional yeasts reveals their potential for aroma formation in food fermentation. Food Microbiol 60: 147–159. 27554157 10.1016/j.fm.2016.07.006

[yea3246-bib-0012] Gibson B , Liti G . 2015 Saccharomyces pastorianus: genomic insights inspiring innovation for industry. Yeast 32: 17–27. 25088523 10.1002/yea.3033

[yea3246-bib-0013] Gibson BR , Storgårds E , Krogerus K , Vidgren V . 2013 Comparative physiology and fermentation performance of Saaz and Frohberg lager yeast strains and the parental species *Saccharomyces eubayanus* . Yeast 30: 255–266. 23695993 10.1002/yea.2960

[yea3246-bib-0014] Goncalves M , Pontes A , Almeida P , *et al* 2016 Distinct domestication trajectories in top‐fermenting beer yeasts and wine yeasts. Curr Biol 26: 2750–2761. 27720622 10.1016/j.cub.2016.08.040

[yea3246-bib-0015] Gonçalves P , Valério E , Correia C , de Almeida JMGCF , Sampaio JP . 2011 Evidence for divergent evolution of growth temperature preference in sympatric *Saccharomyces* species. PLoS One 6: e20739. 10.1371/journal.pone.0020739 PMC3107239 21674061

[yea3246-bib-0016] González SS , Barrio E , Gafner J , Querol A . 2006 Natural hybrids from *Saccharomyces cerevisiae, Saccharomyces bayanus* and *Saccharomyces kudriavzevii* in wine fermentations. FEMS Yeast Res 6: 1221–1234. 17156019 10.1111/j.1567-1364.2006.00126.x

[yea3246-bib-0017] Greig D , Borts RH , Louis EJ , Travisano M . 2002 Epistasis and hybrid sterility in *Saccharomyces* . Proc Biol Sci 269: 1167–1171. 12061961 10.1098/rspb.2002.1989 PMC1691007

[yea3246-bib-0018] Haase SB , Reed SI . 2002 Improved flow cytometric analysis of the budding yeast cell cycle. Cell Cycle 1: 132–136. 12429922

[yea3246-bib-0019] Hahne F , LeMeur N , Brinkman RR , *et al* 2009 flowCore: a bioconductor package for high throughput flow cytometry. BMC Bioinformatics 10: 106. 19358741 10.1186/1471-2105-10-106 PMC2684747

[yea3246-bib-0020] Krogerus K , Magalhães F , Vidgren V , Gibson B . 2015 New lager yeast strains generated by interspecific hybridization. J Ind Microbiol Biotechnol 42: 769–778. 25682107 10.1007/s10295-015-1597-6 PMC4412690

[yea3246-bib-0021] Krogerus K , Magalhães F , Vidgren V , Gibson B . 2016 Ploidy influences the functional attributes of de novo lager yeast hybrids. Appl Microbiol Biotechnol 100: 7203–7222. 27183995 10.1007/s00253-016-7588-3 PMC4947488

[yea3246-bib-0022] Krogerus K , Seppänen‐Laakso T , Castillo S , Gibson B . 2017 Inheritance of brewing‐relevant phenotypes in constructed *Saccharomyces cerevisiae* × *Saccharomyces eubayanus* hybrids. Microb Cell Fact 16: 66. 28431563 10.1186/s12934-017-0679-8 PMC5399851

[yea3246-bib-0023] Lopandic K , Pfliegler WP , Tiefenbrunner W , Gangl H , Sipiczki M , Sterflinger K . 2016 Genotypic and phenotypic evolution of yeast interspecies hybrids during high‐sugar fermentation. Appl Microbiol Biotechnol 100: 6331–6343. 27075738 10.1007/s00253-016-7481-0

[yea3246-bib-0024] López‐Malo M , Querol A , Guillamon JM . 2013 Metabolomic comparison of *Saccharomyces cerevisiae* and the cryotolerant species *S. bayanus* var. *uvarum* and *S. kudriavzevii* during wine fermentation at low temperature. PLoS One 8 e60135. 10.1371/journal.pone.0060135 PMC3603904 23527304

[yea3246-bib-0025] Magalhães F , Vidgren V , Ruohonen L , *et al* 2016 Maltose and maltotriose utilisation by group I strains of the hybrid lager yeast *Saccharomyces pastorianus* . FEMS Yeast Res 16: 380–381. 10.1093/femsyr/fow053 PMC5815069 27364826

[yea3246-bib-0026] Masneuf‐Pomarède I , Bely M , Marullo P , Lonvaud‐Funel A , Dubourdieu D . 2010 Reassessment of phenotypic traits for *Saccharomyces bayanus* var. *uvarum* wine yeast strains. Int J Food Microbiol 139: 79–86. 20188428 10.1016/j.ijfoodmicro.2010.01.038

[yea3246-bib-0027] Mertens S , Steensels J , Saels V , De Rouck G , Aerts G , Verstrepen KJ . 2015 A large set of newly created interspecific *Saccharomyces* hybrids increases aromatic diversity in lager beers. Appl Environ Microbiol 81: 8202–8214. 26407881 10.1128/AEM.02464-15 PMC4651086

[yea3246-bib-0028] Meussdoerffer FG . 2009 A comprehensive history of beer brewing In Handbook of Brewing: Processes, Technology, Markets. Wiley‐VCH: Weinheim; 1–42.

[yea3246-bib-0029] Muir A , Harrison E , Wheals A . 2011 A multiplex set of species‐specific primers for rapid identification of members of the genus *Saccharomyces* . FEMS Yeast Res 11: 552–563. 22093682 10.1111/j.1567-1364.2011.00745.x

[yea3246-bib-0030] Mukai N , Masaki K , Fujii T , Iefuji H . 2014 Single nucleotide polymorphisms of *PAD1* and *FDC1* show a positive relationship with ferulic acid decarboxylation ability among industrial yeasts used in alcoholic beverage production. J Biosci Bioeng 118: 50–55. 24507903 10.1016/j.jbiosc.2013.12.017

[yea3246-bib-0032] Nakao Y , Kanamori T , Itoh T , *et al* 2009 Genome sequence of the lager brewing yeast, an interspecies hybrid. DNA Res 16: 115–129. 19261625 10.1093/dnares/dsp003 PMC2673734

[yea3246-bib-0033] Naumov G , Nguyen H‐V , Naumova E , Michel A , Aigle M , Gaillardin C . 2001 Genetic identification of *Saccharomyces bayanus* var. *uvarum*, a cider‐fermenting yeast. Int J Food Microbiol 65: 163–171. 11393685 10.1016/s0168-1605(00)00515-8

[yea3246-bib-0034] Okuno M , Kajitani R , Ryusui R , Morimoto H , Kodama Y , Itoh T . 2016 Next‐generation sequencing analysis of lager brewing yeast strains reveals the evolutionary history of interspecies hybridization. DNA Res 23: 67–80. 26732986 10.1093/dnares/dsv037 PMC4755528

[yea3246-bib-0035] Paget CM , Schwartz J‐M , Delneri D . 2014 Environmental systems biology of cold‐tolerant phenotype in *Saccharomyces* species adapted to grow at different temperatures. Mol Ecol 23: 5241–5257. 25243355 10.1111/mec.12930 PMC4283049

[yea3246-bib-0036] Pengelly RJ , Wheals AE . 2013 Rapid identification of *Saccharomyces eubayanus* and its hybrids. FEMS Yeast Res 13: 156–161. 23110474 10.1111/1567-1364.12018

[yea3246-bib-0037] Pérez‐Torrado R , Barrio E , Querol A . 2017 Alternative yeasts for winemaking: *Saccharomyces* non‐ *cerevisiae* and its hybrids. *Crit Rev Food Sci Nutr* https://doi.org/10.1080/10408398.2017.1285751 10.1080/10408398.2017.1285751 28362111

[yea3246-bib-0038] Peris D , Moriary RV , Alexander WG , *et al* 2017 Hybridization and adaptive evolution of diverse *Saccharomyces* species for cellulosic biofuel production. Biotechnol Biofuels 10: 78. 28360936 10.1186/s13068-017-0763-7 PMC5369230

[yea3246-bib-0039] Pires EJ , Teixeira JA , Branyik T , Vicente AA . 2014 Yeast: the soul of beer's aroma – a review of flavour‐active esters and higher alcohols produced by the brewing yeast. Appl Microbiol Biotechnol 98: 1937–1949. 24384752 10.1007/s00253-013-5470-0

[yea3246-bib-0040] Richard P , Viljanen K , Penttilä M . 2015 Overexpression of *PAD1* and *FDC1* results in significant cinnamic acid decarboxylase activity in *Saccharomyces cerevisiae* . AMB Express 5: 1–5. 10.1186/s13568-015-0103-x PMC4384992 25852989

[yea3246-bib-0041] Sampaio JP , Gonçalves P . 2008 Natural populations of *Saccharomyces kudriavzevii* in Portugal are associated with oak bark and are sympatric with *S. cerevisiae* and *S. paradoxus* . Appl Environ Microbiol 74: 2144–2152. 18281431 10.1128/AEM.02396-07 PMC2292605

[yea3246-bib-0042] Sebastiani F , Barberio C , Casalone E , Cavalieri D , Polsinelli M . 2002 Crosses between *Saccharomyces cerevisiae* and *Saccharomyces bayanus* generate fertile hybrids. Res Microbiol 153: 53–58. 11881899 10.1016/s0923-2508(01)01286-4

[yea3246-bib-0043] Steensels J , Meersman E , Snoek T , Saels V , Verstrepen KJ . 2014 Large‐scale selection and breeding to generate industrial yeasts with superior aroma production. Appl Environ Microbiol 80: 6965–6975. 25192996 10.1128/AEM.02235-14 PMC4249010

[yea3246-bib-0044] Stratford M , Plumridge A , Archer DB . 2007 Decarboxylation of sorbic acid by spoilage yeasts is associated with the *PAD1* gene. Appl Environ Microbiol 73: 6534–6542. 17766451 10.1128/AEM.01246-07 PMC2075038

[yea3246-bib-0045] Tubb RS , Searle BA , Goodey AR , Brown AJP . 1981 Rare mating and transformation for construction of novel brewing yeasts In Proceedings of the 18th Congress of the European Brewers Convention; 487–496.

[yea3246-bib-0046] Vidgren V . 2010 Maltose and maltotriose transport into ale and lager brewer's yeast strains. VTT Publ 748: 1–158.

[yea3246-bib-0047] Zaret KS , Sherman F . 1985 alpha‐Aminoadipate as a primary nitrogen source for *Saccharomyces cerevisiae* mutants. J Bacteriol 162: 579–583. 3921525 10.1128/jb.162.2.579-583.1985 PMC218887

