# Supplementary material for: Alternative Saccharomyces interspecies hybrid combinations and their potential for low‐temperature wort fermentation
Source: Yeast. 2017 Aug 30;35(1):113–27. doi: 10.1002/yea.3246 (PMC5811906; doi:10.1002/yea.3246)
Supplement: Supplementary file 1 — Table S1. Concentrations (mg L−1) of aroma compounds in the beers produced with the de novo hybrids and parent strains. Values are means from two independent fermentations (standard deviation in parentheses). These values were used to generate the heatmap in Figure 4 in the main text after normalization to ethanol concentration. Table S2. Concentrations (mg L−1) of aroma compounds in the beers produced with the de novo hybrids of different ploidy. Values are means from two independent fermentations (standard deviation in parentheses). These values were used to generate the heatmap in Figure 6 in the main text after normalization to ethanol concentration. Table S3. Concentrations (mg L−1) of aroma compounds in the beers produced with the de novo S. cerevisiae × S. mikatae hybrids and their parent strains. Values are means from two independent fermentations (standard deviation in parentheses). These values were used to generate the heatmap in Figure 8 in the main text after normalization to ethanol concentration. Figure S1. Hybrid confirmation by species‐specific PCR. Strains included are the parental strains S. cerevisiae A81062 and A94132, S. arboricola C15952, S. eubayanus C12902, S. mikatae C15949 and S. uvarum C05774 along with the interspecies hybrids JN2 (A81062 x C15952), H1 (A81062 x C12902), JN10 (A94132 x C15949) and JN5 (A81062 x C05774). Genes amplified are MEX67/YPL169C (S. cerevisiae), SEC24/YIL109C (S. arboricola), FSY1 (S. eubayanus) and DBP6/YNR038W (S. uvarum). All hybrids with the same parental combinations showed identical banding patterns. Figure S2. Growth of the parental strain S. cerevisiae A81062 (Sc), hybrid JN2 (S. cerevisiae A81062 x S. arboricola C15952), hybrid H1 (A81062 x S. eubayanus C12902), hybrid JN7 (A81062 x S. mikatae C15949), and hybrid JN4 (A81062 x S. uvarum C05774). YPD agar cultures were incubated 21 days at 4°C. [file YEA-35-113-s001.pdf]

Supplementary material

Alternative *Saccharomyces* interspecies hybrid combinations and their potential for low-temperature wort fermentation

Yeast

Jarkko Nikulin[1,2], Kristoffer Krogerus[1,3], Brian Gibson[1]*

[1] VTT Technical Research Centre of Finland Ltd, Tietotie 2, P.O. Box 1000, FI-02044 VTT, Espoo, Finland

[2] Chemical Process Engineering, Faculty of Technology, University of Oulu, P.O. Box 8000, FI-90014 Oulun Yliopisto, Finland

[3] Department of Biotechnology and Chemical Technology, Aalto University, School of Chemical Technology, Kemistintie 1, Aalto, P.O. Box 16100, FI-00076 Espoo, Finland

Keywords: *Saccharomyces pastorianus*, lager beer, hybridization, cold tolerance, maltotriose, aroma, phenolic off flavour

*Corresponding author. Tel: +358 207226603, Fax: +358 207227071, Email: brian.gibson@vtt.fi

Table S1. Concentrations (mg L^{-1}) of aroma compounds in the beers produced with the *de novo* hybrids and parent strains. Values are means from two independent fermentations (standard deviation in parentheses). These values were used to generate the heatmap in Figure 4 in the main text after normalization to ethanol concentration.

Compound	Scer A81062	Sarb C15952	Seub C12902	Smik C15949	Suva C05774	Sc × Sa JN2	Sc × Se H1	Sc × Sm JN7	Sc × Su JN4
Acetaldehyde	26.8 (± 2.93)	7.7 (± 1.65)	21.3 (± 1.99)	1.8 (± 0.36)	3.4 (± 2.03)	44.6 (± 14.5)	10.4 (± 0.24)	17.2 (± 4.37)	7.6 (± 0.64)
Propanol	12.5 (± 0.31)	3.2 (± 0.05)	7.2 (± 0.62)	3.4 (± 0.05)	5.0 (± 1.87)	18.2 (± 3.58)	11 (± 0.11)	10.8 (± 0.31)	15.0 (± 1.88)
2-Methylpropanol	7.2 (± 0.27)	2.8 (± 0.04)	12.8 (± 1.09)	2.9 (± 0.05)	4.5 (± 1.89)	9.2 (± 1.83)	11.5 (± 0.22)	8.4 (± 0.05)	12.0 (± 1.69)
3-Methylbutanol	22.0 (± 0.43)	9.5 (± 0.19)	44.0 (± 2.96)	22.4 (± 0.16)	17.2 (± 5.79)	40.4 (± 7.36)	37.3 (± 1.35)	32.3 (± 0.8)	22.8 (± 2.73)
2-Methylbutanol	7.2 (± 0.25)	4.0 (± 0.08)	14.5 (± 1.03)	3.9 (± 0.04)	4.7 (± 0.87)	10.7 (± 2.02)	14.0 (± 0.25)	8.8 (± 0.15)	12.8 (± 1.59)
3-Methybutyl acetate	0.32 (± 0.01)	0.10 (± 0.01)	0.93 (± 0.11)	0.23 (± 0.01)	0.24 (± 0.02)	1.19 (± 0.21)	0.71 (± 0.07)	0.99 (± 0.01)	0.12 (± 0.02)
Ethyl acetate	15.4 (± 0.56)	1.9 (± 0.04)	9.3 (± 1.29)	2.2 (± 0.06)	5.1 (± 3.3)	30.9 (± 6.73)	23.9 (± 1.02)	22.7 (± 1.26)	8.5 (± 1.68)
Ethyl hexanoate	0.21 (± 0.02)	0.08 (± 0.01)	0.11 (± 0.01)	0.04 (± 0.00)	0.12 (± 0.08)	0.26 (± 0.05)	0.30 (± 0.02)	0.42 (± 0.04)	0.10 (± 0.00)
Ethyl octanoate	0.12 (± 0.03)	0.35 (± 0.12)	0.02 (± 0.00)	0.12 (± 0.01)	0.08 (± 0.06)	0.06 (± 0.01)	0.09 (± 0.02)	0.14 (± 0.04)	0.01 (± 0.00)
Ethyl decanoate	0.01 (± 0.001)	0.18 (± 0.078)	0.06 (± 0.015)	0.03 (± 0.005)	0.03 (± 0.008)	0.01 (± 0.005)	0.04 (± 0.009)	0.01 (± 0.003)	ND
2-phenylethanol	11.0 (± 2.44)	8.1 (± 2.08)	45.6 (± 1.91)	13.2 (± 1.31)	12.9 (± 2.72)	10.2 (± 3.90)	14.1 (± 0.55)	15.0 (± 2.0)	11.4 (± 2.3)
2-Phenylethyl acetate	ND	ND	0.92 (± 0.06)	0.17 (± 0.01)	0.17 (± 0.02)	0.14 (± 0.01)	0.19 (± 0.05)	0.24 (± 0.04)	ND

Table S2. Concentrations (mg L^{-1}) of aroma compounds in the beers produced with the *de novo* hybrids of different ploidy. Values are means from two independent fermentations (standard deviation in parentheses). These values were used to generate the heatmap in Figure 6 in the main text after normalization to ethanol concentration.

Compound	Sc × Sm JN6	Sc × Sm JN7	Sc × Su JN4	Sc × Su JN5
Acetaldehyde	4.5 (± 0.40)	14.1 (± 6.21)	11.3 (± 0.27)	5.5 (± 0.16)
Propanol	10.4 (± 0.20)	12.8 (± 0.34)	13.8 (± 0.18)	14.3 (± 0.31)
2-Methylpropanol	9.5 (± 0.19)	8.0 (± 0.34)	13.1 (± 0.41)	12.4 (± 0.45)
3-Methylbutanol	52.7 (± 1.65)	36.8 (± 1.92)	26.0 (± 0.98)	28.4 (± 0.98)
2-Methylbutanol	11.3 (± 0.39)	8.5 (± 0.40)	13.3 (± 0.52)	11.7 (± 0.43)
3-Methybutyl acetate	1.79 (± 0.08)	0.99 (± 0.04)	0.38 (± 0.02)	0.69 (± 0.05)
Ethyl acetate	25.4 (± 0.85)	23.0 (± 0.62)	17.8 (± 0.40)	17.6 (± 0.96)
Ethyl hexanoate	0.35 (± 0.01)	0.34 (± 0.01)	0.18 (± 0.01)	0.24 (± 0.01)
Ethyl octanoate	0.27 (± 0.04)	0.29 (± 0.02)	0.04 (± 0.01)	0.11 (± 0.01)
Ethyl decanoate	0.02 (± 0.008)	0.02 (± 0.001)	0.02 (± 0.004)	0.04 (± 0.007)
2-Phenylethanol	14.6 (± 1.06)	8.6 (± 0.79)	6.2 (± 0.68)	8.0 (± 0.76)
2-Phenylethyl acetate	0.46 (± 0.04)	0.25 (± 0.02)	0.13 (± 0.07)	0.20 (± 0.01)

Table S3. Concentrations (mg L^{-1}) of aroma compounds in the beers produced with the *de novo S. cerevisiae* × *S. mikatae* hybrids and their parent strains. Values are means from two independent fermentations (standard deviation in parentheses). These values were used to generate the heatmap in Figure 8 in the main text after normalization to ethanol concentration.

	Scer A81062	Scer A94132	Smik C15949	Sc × Sm JN7	Sc × Sm JN10	Sc × Sm JN11
Acetaldehyde	11.1 (± 0.82)	4.3 (± 0.65)	0.7 (± 0.09)	5.8 (± 0.42)	2.5 (± 0.15)	1.3 (± 0.16)
Propanol	13.3 (± 0.52)	11.2 (± 1.01)	3.9 (± 0.15)	11.9 (± 0.44)	11.0 (± 0.19)	12.6 (± 0.69)
2-Methylpropanol	5.7 (± 0.04)	5.0 (± 0.35)	3.2 (± 0.04)	7.0 (± 0.33)	8.6 (± 0.24)	7.5 (± 0.08)
3-Methylbutanol	22.4 (± 0.33)	27.9 (± 1.97)	24.5 (± 0.82)	36.8 (± 1.88)	35.5 (± 0.87)	43.2 (± 0.3)
2-Methylbutanol	5.9 (± 0.05)	5.4 (± 0.34)	3.7 (± 0.14)	7.5 (± 0.42)	8.8 (± 0.29)	8.5 (± 0.18)
3-Methybutyl acetate	0.36 (± 0.03)	0.26 (± 0.02)	0.31 (± 0.01)	1.11 (± 0.1)	0.96 (± 0.06)	1.25 (± 0.13)
Ethyl acetate	13.5 (± 0.82)	7.6 (± 0.66)	2.4 (± 0.08)	20.0 (± 1.95)	14.7 (± 0.66)	14.8 (± 1.93)
Ethyl hexanoate	0.27 (± 0.02)	0.23 (± 0.03)	0.07 (± 0.00)	0.37 (± 0.02)	0.27 (± 0.01)	0.37 (± 0.01)
Ethyl octanoate	0.26 (± 0.04)	0.45 (± 0.08)	0.30 (± 0.06)	0.30 (± 0.05)	0.43 (± 0.02)	0.73 (± 0.04)
Ethyl decanoate	0.04 (± 0.008)	0.1 (± 0.028)	0.03 (± 0.011)	0.03 (± 0.005)	0.06 (± 0.002)	0.08 (± 0.016)
2-Phenylethanol	3.8 (± 0.38)	2.6 (± 0.11)	8.5 (± 0.24)	7.4 (± 0.27)	5.1 (± 0.37)	7.7 (± 0.37)
2-Phenylethyl acetate	ND	ND	0.15 (± 0.01)	0.17 (± 0.02)	0.10 (± 0.01)	0.17 (± 0.02)

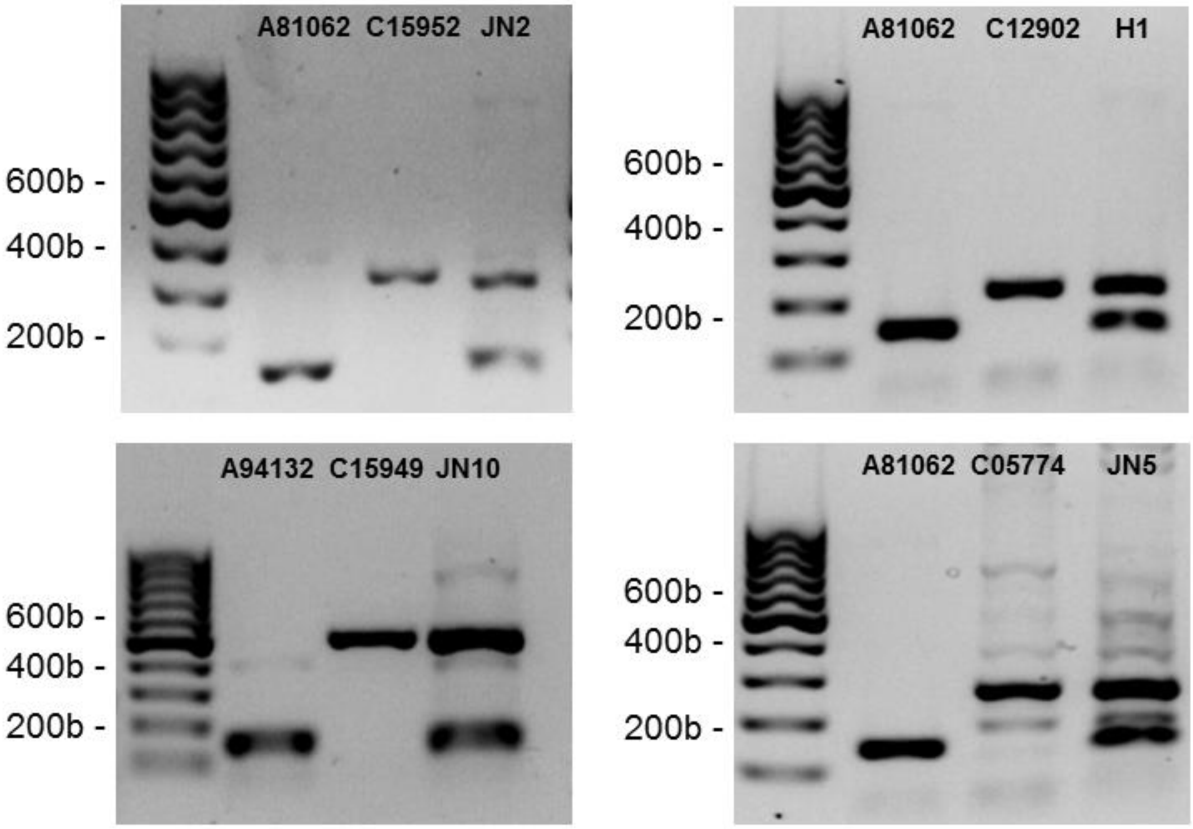

Figure S1. Hybrid confirmation by species-specific PCR. Strains included are the parental strains *S. cerevisiae* A81062 and A94132, *S. arboricola* C15952, *S. eubayanus* C12902, *S. mikatae* C15949 and *S. uvarum* C05774 along with the interspecies hybrids JN2 (A81062 x C15952), H1 (A81062 x C12902), JN10 (A94132 x C15949) and JN5 (A81062 x C05774). Genes amplified are *MEX67*/YPL169C (*S. cerevisiae*), *SEC24*/YIL109C (*S. arboricola*), *FSY1* (*S. eubayanus*) and *DBP6*/YNR038W (*S. uvarum*). All hybrids with the same parental combinations showed identical banding patterns.

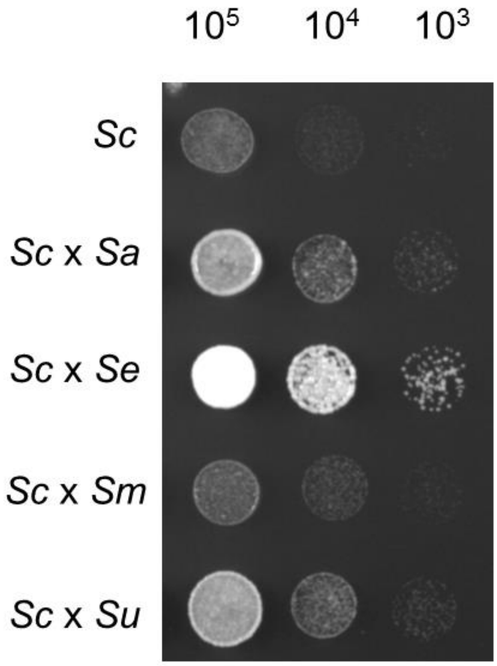

Figure S2. Growth of the parental strain *S. cerevisiae* A81062 (*Sc*), hybrid JN2 (*S. cerevisiae* A81062 x *S. arboricola* C15952), hybrid H1 (A81062 x *S. eubayanus* C12902), hybrid JN7 (A81062 x *S. mikatae* C15949), and hybrid JN4 (A81062 x *S. uvarum* C05774). YPD agar cultures were incubated 21 days at 4°C.
